# Engineered hierarchical 3D scaffold promotes bone regeneration through enhanced mechanotransduction

**DOI:** 10.1093/rb/rbag136

**Published:** 2026-06-17

**Authors:** Dan Wu, Xinyu Dai, Li Xiao, Wenxuan He, Qun Li, Piaoye Ming, Yutao Zhang, Jia Li, Mingxuan Bai, Yuan Wang, Leixiao Yu

**Affiliations:** State Key Laboratory of Oral Diseases & National Center for Stomatology & National Clinical Research Center for Oral Diseases, West China Hospital of Stomatology, Sichuan University, Chengdu, Sichuan 610041, China; State Key Laboratory of Oral Diseases & National Center for Stomatology & National Clinical Research Center for Oral Diseases, West China Hospital of Stomatology, Sichuan University, Chengdu, Sichuan 610041, China; State Key Laboratory of Oral Diseases & National Center for Stomatology & National Clinical Research Center for Oral Diseases, West China Hospital of Stomatology, Sichuan University, Chengdu, Sichuan 610041, China; State Key Laboratory of Oral Diseases & National Center for Stomatology & National Clinical Research Center for Oral Diseases, West China Hospital of Stomatology, Sichuan University, Chengdu, Sichuan 610041, China; State Key Laboratory of Oral Diseases & National Center for Stomatology & National Clinical Research Center for Oral Diseases, West China Hospital of Stomatology, Sichuan University, Chengdu, Sichuan 610041, China; State Key Laboratory of Oral Diseases & National Center for Stomatology & National Clinical Research Center for Oral Diseases, West China Hospital of Stomatology, Sichuan University, Chengdu, Sichuan 610041, China; State Key Laboratory of Oral Diseases & National Center for Stomatology & National Clinical Research Center for Oral Diseases, West China Hospital of Stomatology, Sichuan University, Chengdu, Sichuan 610041, China; State Key Laboratory of Oral Diseases & National Center for Stomatology & National Clinical Research Center for Oral Diseases, West China Hospital of Stomatology, Sichuan University, Chengdu, Sichuan 610041, China; State Key Laboratory of Oral Diseases & National Center for Stomatology & National Clinical Research Center for Oral Diseases, West China Hospital of Stomatology, Sichuan University, Chengdu, Sichuan 610041, China; State Key Laboratory of Oral Diseases & National Center for Stomatology & National Clinical Research Center for Oral Diseases, West China Hospital of Stomatology, Sichuan University, Chengdu, Sichuan 610041, China; State Key Laboratory of Oral Diseases & National Center for Stomatology & National Clinical Research Center for Oral Diseases, West China Hospital of Stomatology, Sichuan University, Chengdu, Sichuan 610041, China

**Keywords:** critical bone defect, hierarchical scaffolds, biointerfaces, bone regeneration, mechano-regulation

## Abstract

Human bone has an innate capacity for self-repair, but it is insufficient to heal critical-sized bone defects. While current scaffolds have the potential to promote the repair of critical-sized defects, this capacity is hindered by their inadequate bioactivity and osteoconductivity. Therefore, a nano-/micro-hierarchical scaffold with a tunable surface architecture was developed to enhance bone regeneration. The resulting biointerface markedly promoted osteogenic differentiation of bone marrow mesenchymal stem cells in a non-linear, roughness-dependent manner. Furthermore, *in vivo* results displayed that scaffolds with a fiber roughness of approximately 400 nm exhibited the highest potency on bone defect repair. The promising performance of the hierarchical scaffold was found to result from enhanced cellular mechanotransduction by its nano-/micro-hierarchical architectures at the scaffold’s biointerface. The present study offers an interesting strategy to address the challenge of critical-sized bone defect repair based on mechanobiology.

## Graphical Abstract

**Graphical Abstract:**
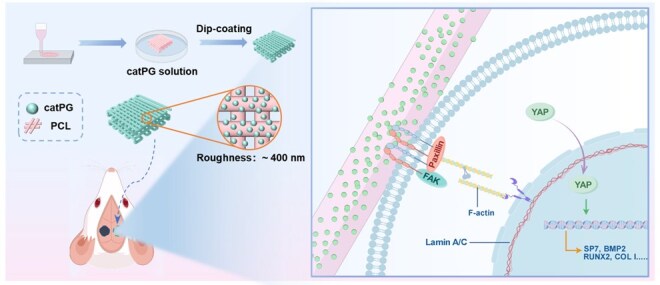

## Introduction

Human bone possesses the innate capacity for self-repair, but this potential is insufficient to heal large-scale injuries, known as critical-sized bone defects, resulting from trauma, tumors, or inflammation [1, 2]. Current clinical treatments primarily rely on autografts or allografts, which are associated with risks including donor site morbidity, unknown donor source, and potential disease transmission [3]. Consequently, repairing critical-sized bone defects remains a significant challenge. Recently, bone tissue engineering has emerged as a promising strategy for bone regeneration [4, 5]. Nevertheless, the limited bioactivity and insufficient osteoconductive capacity of current scaffolds still impede effective repair of critical-sized defects [6, 7].

Bone repair is orchestrated by stem cells residing in skeletal tissues. Upon injury, these cells migrate from the periosteum or bone marrow to the defect site, where they differentiate into osteoblasts and deposit mineralized matrix to facilitate healing [8]. The osteogenic differentiation of stem cells is pivotal for bone regeneration. In addition to the biochemical cues, growing evidence indicates that biophysical cues, e.g. nano-/micro-structures, porosity and matrix stiffness, can regulate stem cell differentiation [9–11]. Among them, surface topography at the biointerface plays a vital role in directing cell behavior through mechanotransduction, especially in promoting osteogenic differentiation of mesenchymal stem cells (MSCs) [12–14]. Mechanotransduction enables cells to convert physical stimuli into biochemical or electrical signals, thereby eliciting physiological responses. Mechanical cues from surface topographies are sensed by mechanosensors on the cell membrane, thus activating the formation of focal adhesions (FAs), reorganizing the cytoskeleton and nucleoskeleton, and ultimately shaping cell morphology and fate specification [15–17]. For instance, surface roughening strategies such as sandblasting with large grits followed by acid etching (SLA) have been widely applied in orthopedic and dental implants, where they significantly enhance osteogenesis and improve implant osseointegration [18]. Although surface modification techniques such as SLA, selective laser melting and anodization are relatively mature, they are often costly and limited in their ability to precisely control surface topography [19]. Therefore, developing a low-cost and controllable strategy for fine surface structuring remains an urgent demand for improving implant performance.

Rather than relying on chemical modifications or exogenous bioactive factors, surface topographical engineering provides a purely biophysical approach to mediate cellular behavior and fate specification. Compared to the biochemical stimuli, biophysical cues offer a stable and non-toxic approach to modulate cell behavior. However, the promotion of osteogenic differentiation by topographical cues is not universal. Bone marrow mesenchymal stem cells (BMSCs) exhibit a non-linear response to surface topography scale. Studies have shown that excessively rough surfaces may inhibit osteogenesis. Only topographies with appropriate roughness effectively support osteogenic differentiation [20]. This scale-dependent effect indicates that ideal bone repair scaffolds should replicate the multiscale architecture of native bone. Notably, native bone is a hierarchically structured and functionally graded tissue. Extracellular matrix (ECM) is highly mineralized, consisting of nanocrystalline hydroxyapatite interwoven with micro-sized collagen fibrils. This unique micro-nano hierarchical structure provides an excellent niche for osteocyte residence and environment inducing bone formation [4, 21, 22].

**Figure 1:**
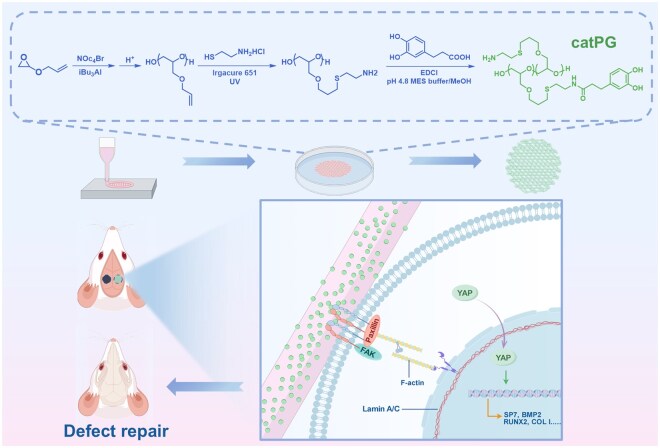
Schematic illustration of nano-/micro-hierarchical scaffolds for bone defect repair through enhanced mechanotransduction pathways. The nano-/micro-hierarchical biointerfaces were constructed by a polyglycerol-based coating polymer, which could *in situ* generate well-defined nanoaggregates on the printed micro-sized PCL fibers.

Inspired by the multilevel structure of natural bone and the regulatory role of surface topography in osteogenesis, an engineered scaffold with tailorable hierarchical surface features was developed herein to facilitate critical-sized bone defect repair. The hierarchical architectures of the scaffold were realized by a catecholic polyglycerol coating polymer (catPG), which rapidly coats the scaffold surface and then slowly but controllably *in situ* crosslinks into nanoparticles and even microaggregates. Leveraging this reactive coating polymer, scaffolds with well-defined nanoparticles homogeneously deposited on the microfibers were effectively obtained by simply dipping the 3D-printed PCL scaffold into the catPG coating solution for several hours. The effects of the resulting scaffolds with hierarchical architectures on BMSC proliferation, adhesion and osteogenic differentiation were systematically investigated. Osteogenesis of BMSC on the scaffold was remarkably enhanced by the presence of nanoparticles but in a non-linear manner. Furthermore, a mechanistic study revealed that the cellular response of BMSCs to the hierarchical architectures occurs through a force-dependent signaling pathway [12, 20]. Both FA formation and Lamin A/C activation were markedly enhanced by these biophysical cues. Thus, the osteogenic differentiation of the cells was correspondingly improved. To validate their bone regeneration performance, a cranial defect model was established, and the *in vivo* bone regeneration capacity of these hierarchical scaffolds was comprehensively assessed. Collectively, scaffolds with hierarchical micro-architectures significantly promoted bone regeneration through enhanced mechanotransduction, offering a promising route for critical-sized bone defect repair (Figure 1).

## Materials and methods

### Preparation of nano-/micro-hierarchical scaffolds

PCL scaffolds (P871874, Macklin, China, Mn = 80 000 g/mol) with a fiber diameter of 250 μm were fabricated using a high-temperature melting 3D printer (EFL, China). The printed PCL scaffolds were placed into a 24-well plate and treated with 1 mL of 1 mg/mL catecholic polyglycerol coating polymer in a mixture of methanol (MeOH) and 3-(N-morpholino)-propanesulfonic acid (MOPS, 0.1 M, pH 8.6) buffer (v/v 3:1) at room temperature for several hours. The biomimetic scaffolds were then washed sequentially with methanol and Milli-Q water, dried and placed in a UV sterilizer. Prior to experimentation, all scaffolds were sterilized using ultraviolet light for 30 min.

### Characterization of scaffolds

The surface morphology of the scaffolds was observed using scanning electron microscopy (SEM, Hitachi, Japan). Surface roughness was measured with confocal laser scanning microscopy (CLSM, Zeiss, Germany). Elemental composition was analyzed by energy-dispersive X-ray spectroscopy (EDS). Hydrophilicity was determined using an optical contact angle (CA) goniometer.

### Cell culture

BMSCs were isolated from the femurs of 10-day-old Sprague–Dawley rats and cultured in α-MEM medium (PM150421, Pricella, China) supplemented with 10% fetal bovine serum (A5669701, Gibco, USA) and 1% penicillin/streptomycin (15140-122, Gibco, USA). Cells were maintained in T25 culture flasks and incubated at 37°C with 5% CO_2_. Cells at passage 3–5 were used in subsequent experiments.

### Cell viability and proliferation

For cell viability assessment, scaffolds were placed into 48-well plates, and 1 × 10^5^ cells were seeded per well. After 1 and 3 days of culture, cells were washed with phosphate buffered saline (PB180327, Pricella, China) and stained with a Calcein-AM/PI staining kit (C2015M, Beyotime, China). Live and dead cells were observed under CLSM (Leica, Germany). To evaluate cell proliferation, scaffolds were placed into 96-well plates, and 1 × 10^4^ cells were seeded per well. After 1, 3 and 7 days, 10% CCK-8 reagent (BS350A, Beyotime, China) was added to each well, and cells were incubated at 37°C for 1 h in the dark. Absorbance at 450 nm was measured using a microplate reader (Thermo, USA).

### Cell adhesion

Scaffolds were placed into 24-well plates, and 2 × 10^5^ cells were seeded per well. After 24 h of culture, cells were washed with PBS, fixed in 4% paraformaldehyde (BL539A, Biosharp, China) for 20 min, and then washed three times with PBS. Cells were permeabilized with 0.1% Triton X-100 (P0096, Beyotime, China) for 10 min, and the cytoskeleton was stained overnight at 4°C with Phalloidin-FITC (KTC4008, Abbkine, China). Nuclei were stained with Hoechst 33342 (H4079, UElandy, China) for 10 min at room temperature. After three PBS washes, cells were treated with an anti-fluorescence quenching agent (P0126, Beyotime, China). Cell morphology was observed using CLSM (Olympus, Japan). For SEM observation, cells were fixed with 2.5% glutaraldehyde, washed with PBS, and dehydrated through an ethanol gradient (30%, 50%, 70%, 85%, 95%, 100%). Hexamethyldisilazane was used to remove residual water.

### Immunofluorescence staining

After 24 h of cell seeding, cells were fixed in 4% paraformaldehyde for 20 min. They were then washed three times with PBS and permeabilized with 0.1% Triton X-100 for 10 min. After PBS washing, cells were blocked with 5% bovine serum albumin (4240GR025, Bioforxx, Germany) overnight at 4°C. Primary antibodies were added and incubated overnight at 4°C. The primary antibodies used included Paxillin (610051, BD, USA), p-FAK (700255, Invitrogen, USA), Lamin A/C (10298-1-AP, Proteintech, China) and Yes-associated protein (YAP) (WL03624, Wanleibio, China). After three PBS washes, cells were incubated for 1 h at room temperature with appropriate secondary antibodies (Jackson ImmunoResearch, USA). The nuclei were stained with Hoechst 33342. An anti-fluorescence quenching agent was applied after PBS washing, and fluorescence imaging was performed using CLSM.

### Osteogenic differentiation

After 24 h of cell seeding, the culture medium was replaced with osteogenic differentiation medium, with media changes every 3 days. After 7 days, cells were fixed in 4% paraformaldehyde and stained using the BCIP/NBT ALP color development kit (C3206, Beyotime, China). After stopping the reaction, images were taken using an inverted microscope (Olympus, Japan). After 28 days, cells were fixed in 4% paraformaldehyde, and Alizarin Red S (ARS) staining (C0148S, Beyotime, China) was performed. After removing excess dye with PBS, images were captured under an inverted microscope. Calcium nodules were dissolved using 10% cetylpyridinium chloride, and absorbance was measured at 562 nm using a microplate reader.

### Establishment of rat cranial defect model

All animal experiments were approved by the Animal Experiment Center of West China Hospital, Sichuan University (approval no.: 20241015004). In this study, 6-week-old male Sprague–Dawley rats (approximately 200 g, Dossy, China) were used for *in vivo* bone defect regeneration studies. The rats were divided into four groups: Blank, RG0, RG-400 and RG-1000. After anesthesia, hair shaving and disinfection, an incision was made to expose the periosteum and calvaria. Two symmetrical cranial defects (5 mm in diameter) were created on either side of the sagittal suture using a trephine. Biomimetic scaffolds from each group were implanted into the defect areas, and the wounds were sutured. Four and 8 weeks after scaffold implantation, the rats were euthanized, and cranial samples were harvested and fixed in 4% paraformaldehyde. After 48 h, the samples were transferred to PBS.

### Micro-computed tomography and histological analysis

New bone formation in the cranial defect area was evaluated using a μ-computed tomography (CT) imaging system (SCANCO, μCT 45, Switzerland). The region of interest in the defect area was selected, and 3D reconstructions of the calvaria were generated. Data such as bone volume/total volume (BV/TV) for each sample were calculated. After decalcification using 17% EDTA (solution changed every 3 days for 1 month), the samples were dehydrated, embedded in paraffin and sectioned into 5-μm-thick slices using a microtome. Sections were stained with H&E, Masson’s trichrome, immunohistochemical and immunofluorescence stains.

### Statistical analysis

All data are presented as mean ± standard deviation, with at least three biological replicates per experiment. Comparisons among multiple groups were conducted using one-way analysis of variance (ANOVA) followed by Tukey's multiple comparison test. For data that did not meet the assumptions of normal distribution, including cell spreading area, filopodia length, and FAs area, statistical significance was evaluated using the Kruskal–Wallis test followed by Dunn’s multiple comparisons test. A p-value of less than 0.05 was considered statistically significant (*p < 0.05, **p < 0.01, ***p < 0.001, ****p < 0.0001).

## Results and discussion

### Preparation of nano-/micro-hierarchical scaffolds

**Figure 2:**
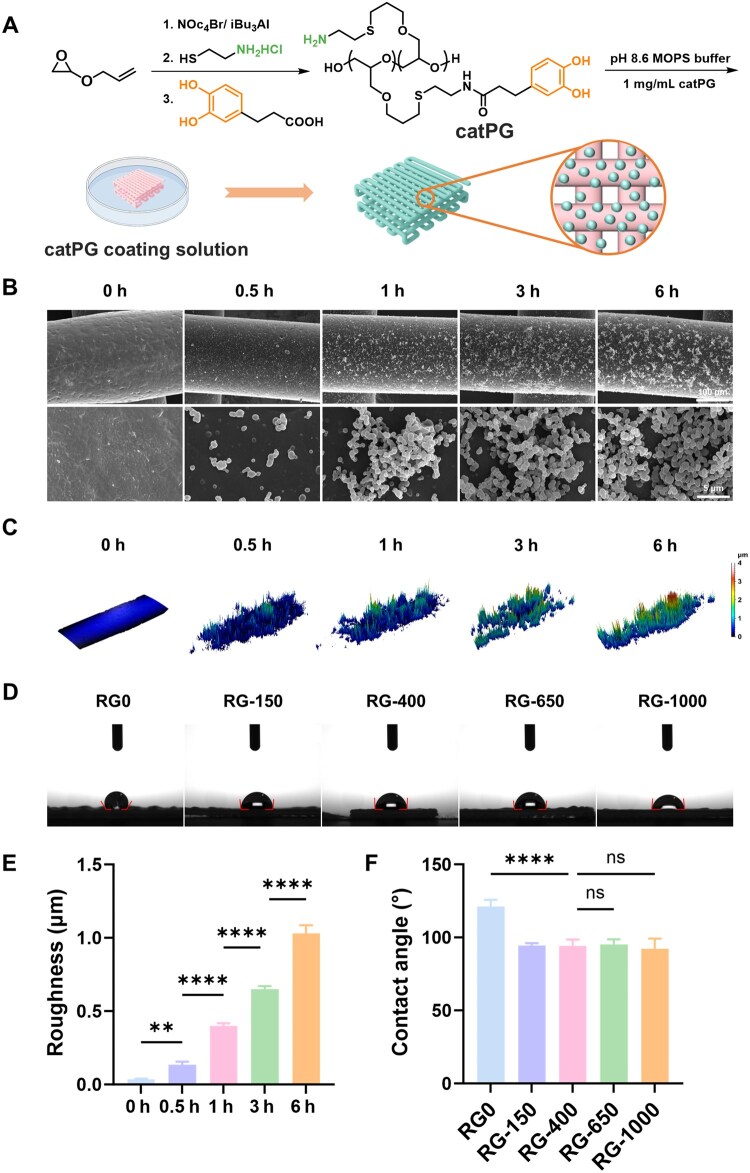
Characterization of the hierarchical scaffolds. (**A**) Schematic illustration of the preparation of catPG coating polymer and the fabrication of hierarchical scaffolds. (**B**) SEM images of the surface architecture of the coated PCL scaffold fibers in a broad (upper) and zoomed-in version (bottom). The scale bars indicate 100 and 5 µm respectively. (**C**) CLSM images of the surface morphology of coated PCL scaffold fibers in 3D-model. (**D**) Static water CAs of the coated scaffolds. (**E**) Average surface roughness (*R*_a_) of the coated PCL scaffold fiber based on CLSM images (*n* = 3). (**F**) Water CAs of the coated scaffold surface (*n* = 3). (*p < 0.05, **p < 0.01, ***p < 0.001, ****p < 0.0001).

To mimic the nano-/micro-hierarchical biointerface of natural bone, a catPG was developed to *in situ* generate well-defined nanoaggregates on 3D-printed PCL fibers, resembling the hydroxyapatite assemblies on collagen fibers in natural bone. The catPG coating polymer was synthesized via the ring-opening anionic polymerization of allyl glycidyl ether, followed by thio-ene amination with cysteamine and amide coupling with 3-(3,4-dihydroxyphenyl)-2-hydroxypropanoic acid (DHHA) (Figure 2A). The catechol grafting density was controlled by tuning the equivalent ratio between DHHA and amino groups in the polymer chain. The obtained catPG was characterized by ^1^H-NMR (Supplementary Figure S3). The catechol grafting density in catPG was 41%, calculated from the integrals of catechol and cysteamine peaks from ^1^H-NMR spectra.

Herein, catPG utilizes catechol moieties as anchoring sites, while amine groups provide reactive centers for intra- and intermolecular crosslinking. The interplay between catecholic oxidation and amine-induced crosslinking forms a stable and robust network [23]. Under basic conditions, catPG undergoes covalent crosslinking and self-aggregation into partially insoluble nanoparticles in the coating solution (methanol/water mixture). The nanoparticles are subsequently deposited onto the surface of 3D-printed PCL scaffolds (Figure 2A and B). Benefiting from the abundance of reactive catechol and amine groups, the deposited particles progressively grow via consecutive Michael addition and dopa–quinone coupling [24]. The coating kinetics could be controlled by adjusting the buffer pH and polymer concentration. The thickness and roughness of the resulting coating layer were dependent on the incubation time.

Generally, the 3D-printed PCL scaffold was immersed in catPG solution (1 mg/mL, methanol/pH 8.6 MOPS buffer = 3:1) at room temperature. As shown in Figure 2B, the catPG nanoaggregates were gradually deposited and uniformly covered the PCL microfibers. The coverage and thickness of the coating layer progressively increased with prolonged incubation. EDS elemental mapping of hierarchical scaffolds after 24-h dip-coating confirmed the successful deposition of catPG on the scaffold surface (Supplementary Figure S4). The morphology and average roughness (Ra) of coatings formed at different incubation times were further characterized using CLSM (Figure 2C). With increasing incubation time, catPG particles on PCL fibers progressively grow from nano- to microaggregates. Correspondingly, the average surface roughness of the coated PCL fiber increases from 150 nm (RG-150: 0.5-h incubation) to 1030 nm (RG-1000: 6-h incubation) (Figure 2E). Scaffolds coated for different durations were thus termed as RG-150, RG-400, RG-650 and RG-1000 according to their surface roughness values. The scaffold without catPG coating was termed RG0. Compared with our previously reported mussel-inspired coating polymer (MI-dPG) with dendritic architecture [12, 20, 25], the linear catPG exhibited a larger hydrodynamic radius and higher chain entanglement [24, 26]. Therefore, the reaction kinetics of heteromultivalent catechol and amine groups on the linear catPG were slower than those of the dendritic polymer, resulting in a slower yet better-controlled coating process [12, 20].

Surface wettability is a key factor that affects cell adhesion and spreading [27]. The CA of the catPG-coated scaffolds were therefore measured. As shown in Figure 2D, all the coated scaffolds displayed an increased hydrophilicity compared to the untreated scaffolds. The CA of the coated scaffolds was about 92°, while it was ∼121° for untreated PCL scaffolds. It has been recognized that the wettability of a surface depends not only on the surface chemical composition but also on the surface architecture. Roughness plays an important role in surface static CA [28]. However, there was no significant difference in CA among the coated scaffolds with varying fiber roughness (Figure 2F). This is because the measured surface static CA reflect the overall scaffold wettability rather than that of individual PCL fibers. Although fiber roughness increased with catPG deposition, the average surface roughness of the entire 3D scaffold remained largely unchanged due to the porous, layered architecture. The increased hydrophilicity was, therefore, mainly attributed to the introduction of amine-rich catPG coatings.

### Nano-/micro-hierarchical scaffolds mediate cell adhesion and spreading

**Figure 3:**
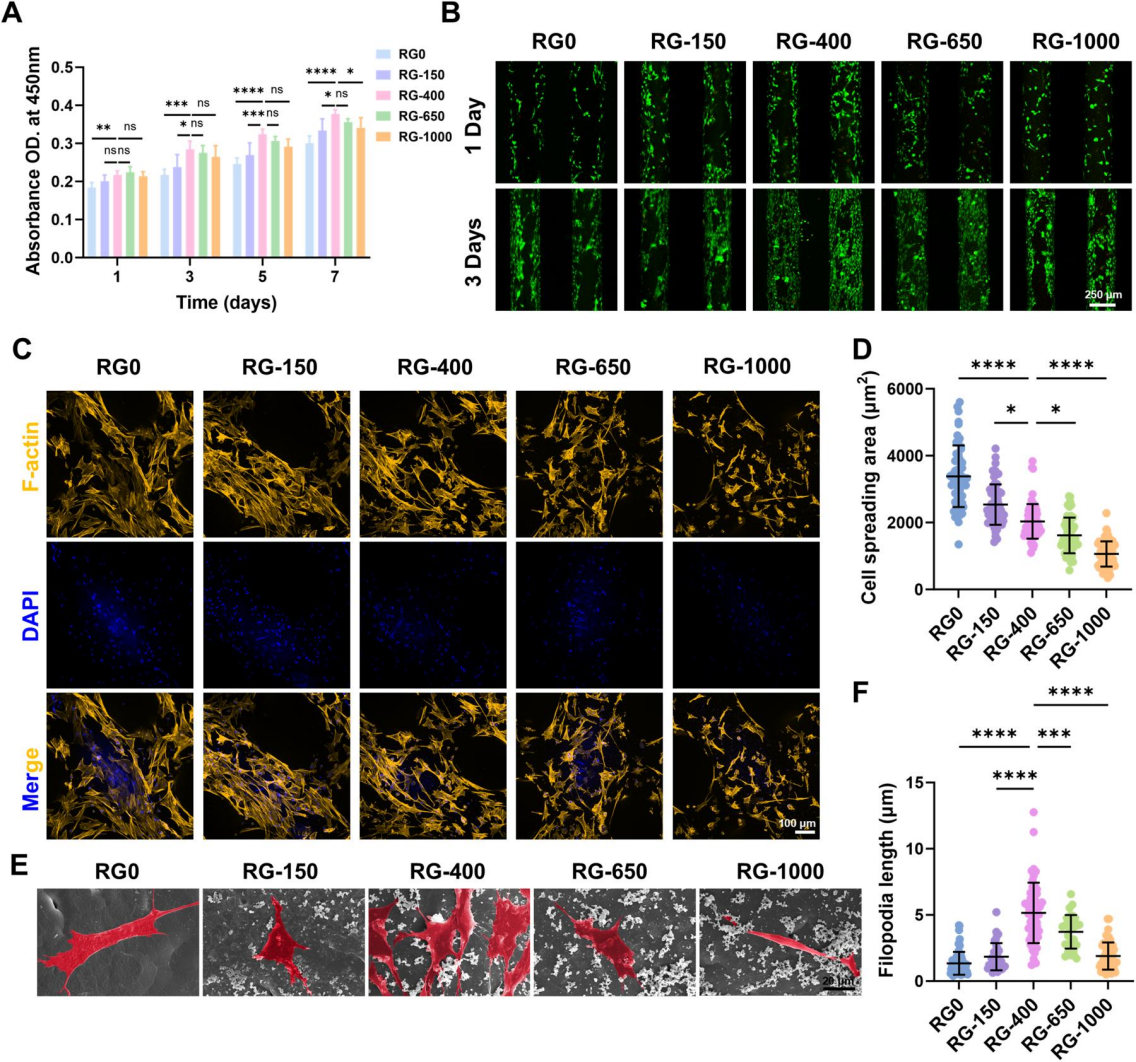
Cell adhesion and spreading on coated scaffolds. (**A**) Proliferation of BMSCs on the scaffolds analyzed through the CCK-8 kits (*n* = 6). (**B**) Live/dead staining of BMSCs attached onto the scaffolds. Scale bar indicates 250 µm. (**C**) Fluorescent staining of actin filaments in BMSCs after 24 h of culture on the scaffolds. Scale bar indicates 100 µm. (**D**) Quantification of cell spreading area after 24 h of culture on the scaffolds (*n* = 55). (**E**) SEM images of BMSCs adhered to the scaffold surfaces. Scale bar indicates 20 µm. (**F**) Filopodia length of the adhered BMSCs (*n* = 30). (*p < 0.05, **p < 0.01, ***p < 0.001, ****p < 0.0001).

To investigate cell proliferation on the nano-/micro-hierarchical scaffolds, BMSCs were co-incubated with the scaffolds and analyzed through the CCK8 assay. As shown in Figure 3A, BMSCs exhibited markedly enhanced proliferation on catPG-coated scaffolds compared with the untreated scaffold (RG0). The presence of nanoaggregates on the scaffold fiber facilitated the attachment and growth of BMSCs. Interestingly, the enhancement in cell proliferation exhibited a fiber-roughness-dependent but non-linear trend. After being cocultured for 1 day, cells on the RG-400 scaffold exhibited the highest proliferative capacity among all groups. These results are consistent with previous reported observations of cell behavior on rough surfaces [12, 20]. Cell attachment and proliferation on the coated scaffolds were further visualized by live/dead staining. As shown in Figure 3B, the cell numbers on the scaffolds maintained a similar trend to the CCK8 results. These results further confirmed that the scaffold fiber architecture plays a significant role in regulating cell proliferation.

Furthermore, the effects of nano-/micro-architectures on cell adhesion and spreading were examined. BMSCs were seeded on the coated scaffolds and cultured for 24 h, with untreated scaffolds serving as the control. As shown in Figure 3C and D, the cellular spreading area gradually decreased as the fiber roughness increased. Cell spreading in the 2D plane was restricted by the nanoaggregates on the fiber surface, whereas the coated scaffolds still retained an open 3D porous architecture that allowed space for vertical extension. As previously reported, the surface features at biointerfaces may act as “energy barriers” to disrupt cell membrane extension in the horizontal direction [20]. In such case, cells tend to adjust their adhesion orientation, extending into the scaffold pores to increase the contact area in the vertical direction [29].

Filopodia are actin-rich membrane protrusions crucial for sensing the biointerface and guiding cell adhesion and migration in both 2D and 3D environments [30, 31]. To further evaluate cell–scaffold interactions, filopodia formation on the hierarchical scaffolds was analyzed (Figure 3E). Both the number and length of filopodia increased with surface roughness and peaked on the RG-400 scaffolds. For scaffolds with higher fiber roughness (RG-650 and RG-1000), the enhancement of filopodia formation tended to decrease. However, filopodia length on RG-1000 remained slightly higher than that on the uncoated scaffold (RG0) (Figure 3F). These results indicate that although the nanoaggregates restricted lateral cell spreading, they promoted vertical extension by stimulating filopodia-mediated anchoring. However, when aggregate size exceeded a certain threshold, the enlarged aggregates acted as “energy barriers,” hindering membrane extension and invasion in both horizontal and vertical directions. Consequently, filopodia formation was also partially suppressed. Overall, these results suggest that moderate surface roughness promotes cell adhesion and proliferation by balancing lateral confinement and vertical engagement. Such behavior aligns with previously reported findings about cell spreading and adhesion on biointerfaces with discontinuous or irregular geometrical features [29, 32].

### Cells mechanically sense and respond to the scaffold’s hierarchical biointerface

**Figure 4:**
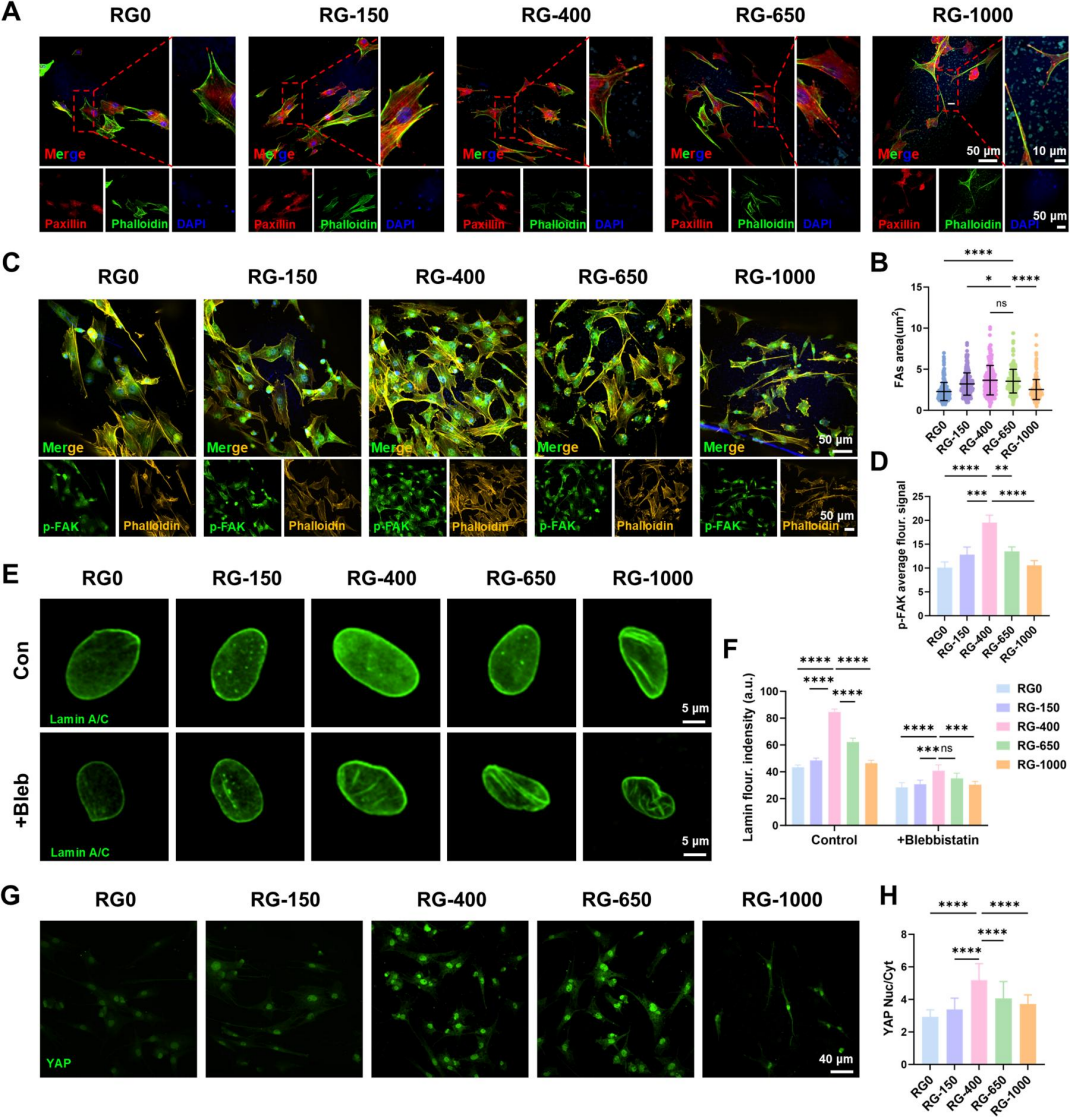
Cells sense hierarchical architectures on scaffold surface through FAs and nuclear mechanics. (**A**) Representative immunofluorescence staining images of paxillin in BMSCs cultured on coated scaffolds for 24 h with the untreated scaffolds as control. (**B**) Quantitative analysis of the focal adhesion area (*n* = 240). (**C**) Representative immunofluorescence staining images of p-FAK in BMSCs cultured on coated scaffolds for 24 h with the untreated scaffolds as control. (**D**) Relative average fluorescent intensity of p-FAK immunostaining for BMSCs (*n* = 3). (**E**) Representative immunofluorescence images of Lamin A/C showing nuclear morphology (upper) and representative immunofluorescence images of Lamin A/C for BMSCs after 24 h of blebbistatin treatment (bottom). (**F**) Quantitative analysis of Lamin A/C fluorescence intensity and quantitative analysis of Lamin A/C fluorescence intensity in BMSCs after 24 h of blebbistatin treatment (*n* = 6). (**G**) Representative immunofluorescence images of YAP for BMSCs. (**H**) YAP nuclear/cytoplasmic ratio for YAP nuclear translocation analysis (*n* = 30). (*p < 0.05, **p < 0.01, ***p < 0.001, ****p < 0.0001).

To elucidate how cells sense and respond to the hierarchical biointerface of coated scaffolds, the formation and mechanotransductive roles of FAs were investigated. FAs are protein complexes that physically link the actin cytoskeleton to ECM via integrins [33–35]. The recruitment of paxillin and phosphorylation of focal adhesion kinase (FAK) are widely recognized as molecular indicators of FA assembly and maturation [36, 37]. Therefore, paxillin localization and FAK phosphorylation were examined in BMSCs cultured on scaffolds for 24 h. Paxillin-positive FAs were observed on all scaffold surfaces, and their area progressively increased with fiber roughness and peaked on the RG-400 group, whereas it decreased on RG-1000 scaffolds, approaching the level observed on uncoated controls (RG0) (Figure 4A and B). FA area exhibited a dependence on surface roughness similar to that observed for cell proliferation and filopodia development.

Phosphorylation of FAK positively correlates with the intracellular traction forces and mechanotransduction efficiency [37–40]. Consistent with FA quantification, the p-FAK intensity displayed a biphasic correlation with scaffold roughness (Figure  4C and D). Cells on RG-400 scaffolds exhibited maximal p-FAK intensity, while higher roughness (RG-650 and RG-1000) suppressed intracellular traction force generation.

Lamin A/C, a structural protein of the nuclear envelope, is involved in mechanical force transmission by connecting to actin filaments to provide mechanical support [34, 41, 42]. Higher Lamin A/C expression typically reflects increased nuclear tension and mechanotransductive activity. As shown in Figure 4E, cells on uncoated and moderately rough scaffolds (RG-150, RG-400 and RG-650) exhibited smooth and extended nuclear envelopes, whereas those on RG-1000 displayed wrinkled and collapsed nuclei. Quantitative analysis of Lamin A/C fluorescence confirmed that moderate roughness enhanced Lamin A/C expression, while excessive roughness downregulated it (Figure 4F). Generally, mechanical forces flatten the nucleus and upregulate Lamin A/C via traction forces transmitted through the LINC complex [43–45]. Thus, the nanoscale aggregates on scaffold fibers effectively increased cytoskeletal tension and enhanced mechanotransduction.

To further verify whether the Lamin A/C expression was regulated by myosin-induced mechanotransduction, BMSCs cultured on the scaffolds were treated with a low dose of blebbistatin for 24 h. Blebbistatin is a small molecule that inhibits the activity of myosin II. Myosin II is the major motor protein responsible for the generation of cytoskeletal tension and is crucial for mechanotransduction pathways [46]. Upon treatment with blebbistatin, Lamin A/C expression markedly decreased across all scaffold groups, and nuclear envelopes became more contracted and wrinkled (Figure 4E and F). Inhibition of actomyosin contractility would diminish nuclear tension and reduce nuclear resistance to external forces. Therefore, the extent of nuclear membrane wrinkling following blebbistatin treatment correlated positively with scaffold roughness, confirming that cellular mechano-sensing and nuclear mechanics on these hierarchical scaffolds are myosin-dependent.

YAP is a key mechano-transducer that shuttles between the cytoplasm and nucleus to activate TEAD-mediated transcription in response to mechanical stimuli [47–52]. As shown in Figure 4G, total YAP expression increased in cells cultured on coated scaffolds with low-to-moderate roughness (RG-150, RG-400 and RG-650), where prominent nuclear localization was observed. Quantification of the nuclear-to-cytoplasmic YAP ratio indicated that cells on RG-400 scaffolds exhibited the highest level of YAP nuclear translocation (Figure 4H).

Collectively, these findings demonstrated that hierarchical scaffolds with optimal nanoscale roughness promote efficient FA formation and FAK activation, leading to enhanced cytoskeletal tension and downstream nuclear mechanotransduction, including Lamin A/C regulation and YAP nuclear translocation. In contrast, excessively rough microscale fiber architectures acted as physical impediments, attenuating FA assembly and hindering mechanotransductive signaling.

### Nano-/micro-hierarchical scaffolds enhance osteogenesis of BMSCs

**Figure 5:**
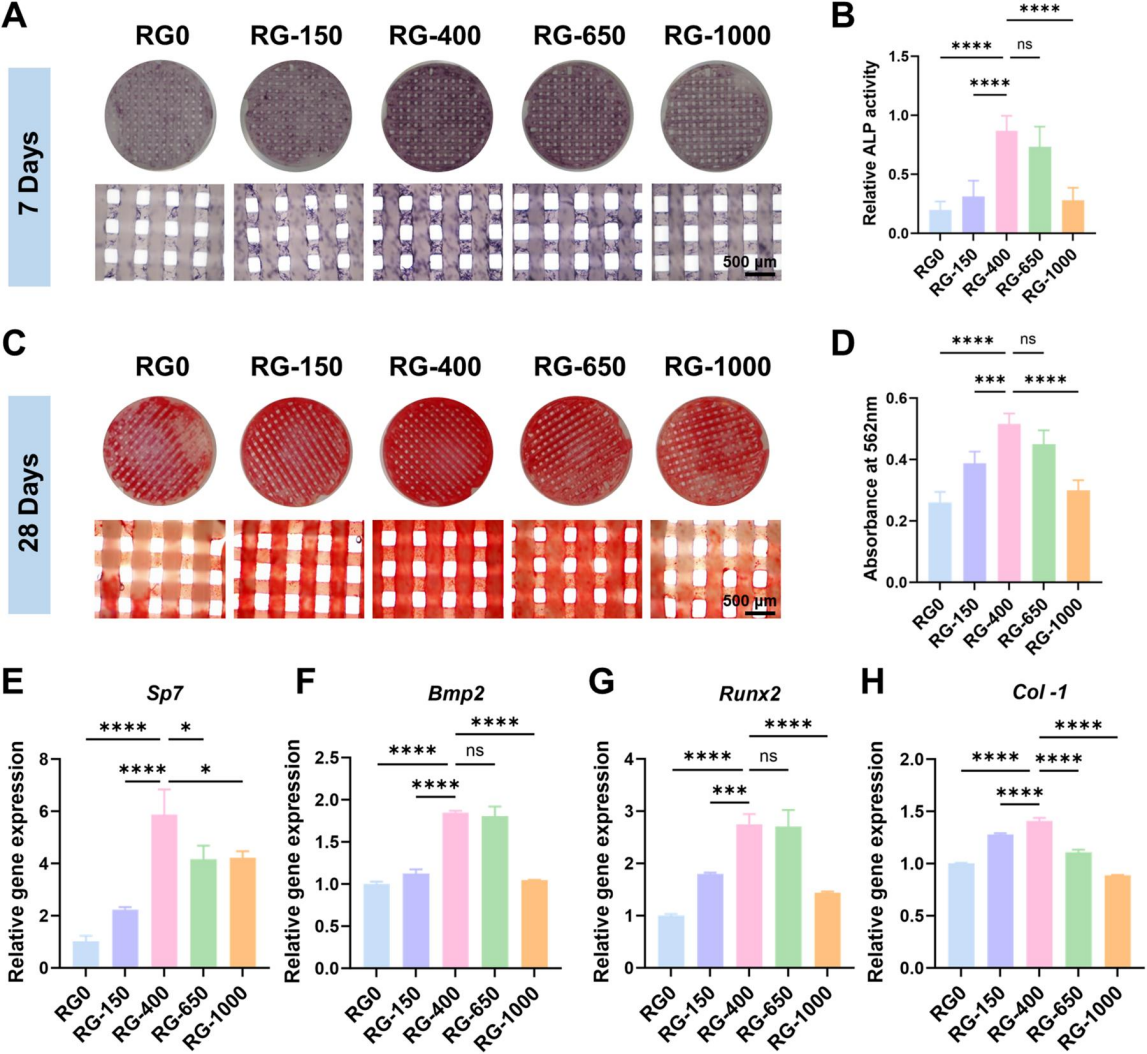
Hierarchical scaffolds promote osteogenic differentiation of BMSCs. (**A**) ALP staining images of BMSCs cultured on scaffolds for 7 days. (**B**) Quantitative analysis of relative ALP activity (*n* = 5). (**C**) ARS staining images of BMSCs cultured on scaffolds for 28 days. (**D**) Quantitative analysis of ARS staining (*n* = 5). (**E**) qRT-PCR analysis of *Sp7* gene expression changes and osteogenic-related genes including (**F**) *Bmp2*, (**G**) *Runx2* and (**H**) *Col-1* in BMSCs cultured on scaffolds for 7 days (*n* = 3). (*p < 0.05, **p < 0.01, ***p < 0.001, ****p < 0.0001).

Osteoinductivity is an essential property required for bone tissue scaffolds. As discussed above, the coated scaffolds with nanoscale hierarchical architectures markedly enhanced the nuclear mechanics and YAP nuclear translocation, both of which are known to promote the osteogenic differentiation of BMSCs [53]. Therefore, the osteoinductive capacity of the hierarchical scaffolds was comprehensively evaluated through alkaline phosphatase (ALP) staining, ARS staining and expression analysis of osteogenesis-related genes. After 7 days of culture in osteogenic differentiation medium, BMSCs were stained with ALP kits. As shown in Figure 5A, a significant increase in ALP activity was observed on hierarchical scaffolds with nanoscale rough fiber architectures compared to both uncoated scaffolds and those with microscale roughness. Among them, RG-400 group displayed the most pronounced ALP activity (Figure 5B). In addition to ALP activity, calcium nodule formation is also a classical marker used to assess osteogenesis, which was further evaluated through ARS staining after 28 days of culture. Consistent with ALP results, abundant and dense mineralized nodules were evident on scaffolds with suitable fiber roughness (Figure 5C and D), with the highest mineral deposition found in the RG-400 group. These results demonstrate that the nanoscale aggregates present on scaffold fibers strongly enhance the osteogenic differentiation of BMSCs.

To further confirm the osteoinductive effects, quantitative real-time polymerase chain reaction (qRT-PCR) was performed to analyze the expression of osteogenesis-related genes (Figure 5E–H). *Sp7* transcription factor (*Sp7*) is a key transcription factor involved in osteoblast differentiation and bone formation [54, 55]. As shown in Figure 5E, cells cultured on the scaffolds with nanoscale rough fibers exhibited significant *Sp7* upregulation, as its expression level was non-linearly dependent on the fiber roughness. The expression level of other osteogenesis-related genes, including Collagen Type I (*Col-1*), Bone Morphogenetic Protein (*Bmp2*) and Runt-Related Transcription Factor 2 (*Runx2*), showed similar upregulation trends in BMSCs cultured on catPG-coated scaffolds. Subsequently, to investigate the role of YAP-mediated mechanotransduction during osteogenic differentiation, cells were treated with Verteporfin (a YAP inhibitor) during osteogenic induction, followed by qRT-PCR analysis. After Verteporfin treatment, the expression of osteogenesis-related genes in cells cultured on the RG-400 scaffold was significantly downregulated (Supplementary Figure S6). Overall, RG-400 scaffolds with nanoscale fiber roughness exhibited the highest osteoinductive activity, which may result from the highest cytoskeletal tension, nuclear mechanics and YAP nuclear translocation.

Several studies have suggested that catechol and amino functional groups may promote osteogenic differentiation [56–59]. However, in the present system, these groups are largely incorporated into nanoparticle-like deposits formed on the scaffold surface, which may limit their effective exposure to cells and thus reduce their direct biological contribution.

Consistently, although the RG-1000 group contains a higher amount of deposited catPG, its osteogenic performance is reduced, indicating that surface chemistry alone cannot account for the observed trends. In addition, our previous work has shown that even when surface chemical differences are minimized, cell adhesion and spreading behaviors remain primarily governed by underlying topographical features [12]. This trend further indicates that topography-mediated physical interactions, rather than surface chemistry, play a dominant role in regulating cellular behavior and mechanotransduction in the present system.

### Nano-/micro-hierarchical scaffolds promote critical cranial defect repair

**Figure 6:**
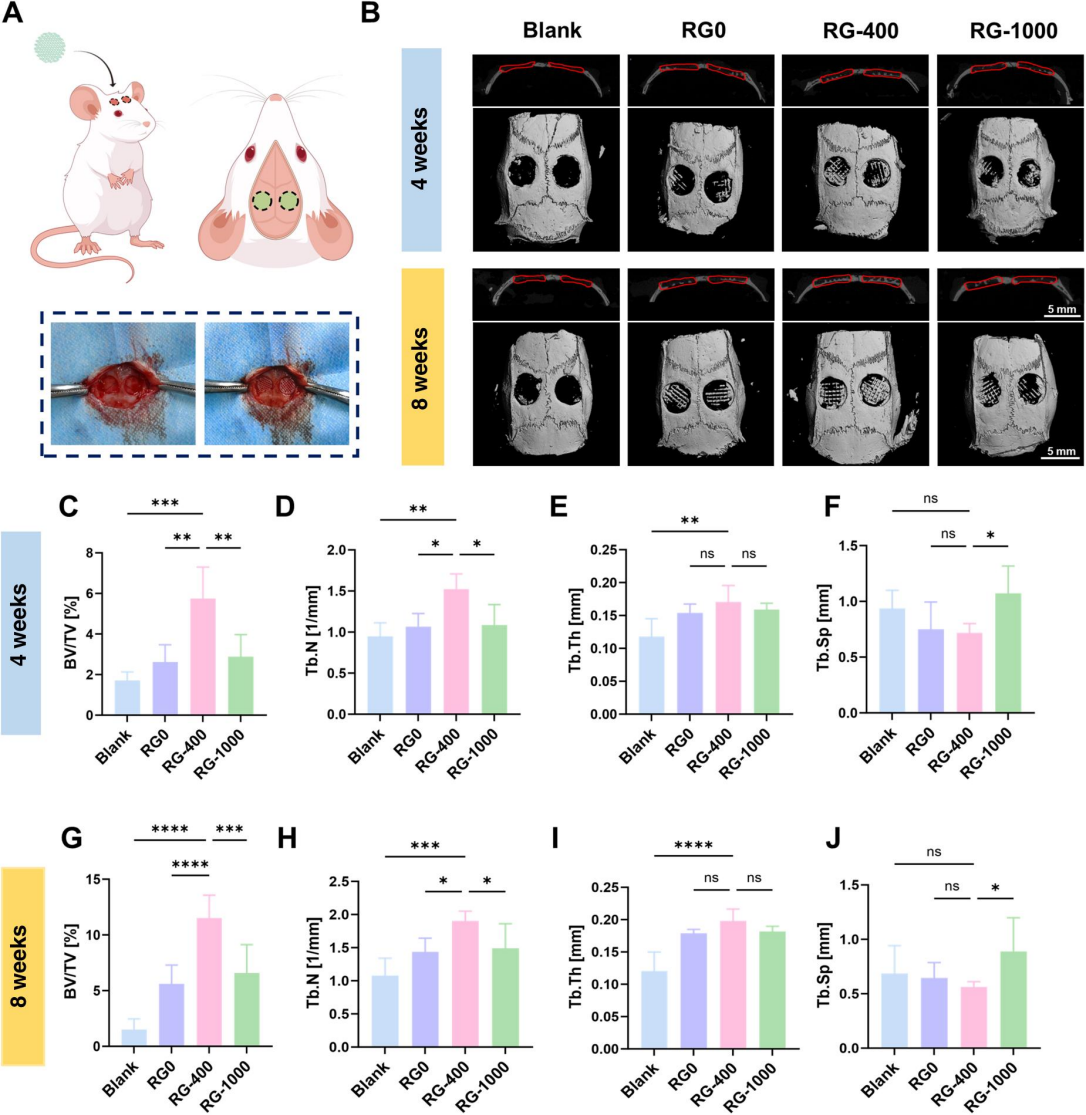
Hierarchical scaffolds promote critical bone defect repair. (**A**) Schematic illustration of scaffold implantation in a critical-sized cranial defect model. (**B**) Representative micro-CT reconstruction volume images of the defects at 4 and 8 weeks post-implantation (*n* = 5). (**C**–**J**) quantitative analysis of BV/TV, Tb.N, Tb.Th, and Tb.Sp of new bone in the defect area at 4 and 8 weeks (*n* = 5). (*p < 0.05, **p < 0.01, ***p < 0.001, ****p < 0.0001).

To evaluate the *in vivo* bone regeneration capability of the hierarchical scaffolds, a critical-sized cranial defect model was established in rats. Based on the previous *in vitro* results, the RG-400 hierarchical scaffold, representing the optimal nanoscale roughness condition, and the RG-1000 scaffold, representing an excessively rough condition, were selected for implantation into the bone defects to evaluate the roughness-dependent osteogenic response *in vivo*. The uncoated RG0 scaffold was included as the material control, while defects without scaffold implantation served as the blank control group (Figure 6A). Rats were euthanized at 4 and 8 weeks after implantation, and new bone formation within the defect area was assessed by micro-computed tomography (micro-CT). As shown in Figure 6B, the newly formed bone could be clearly observed in all scaffold-implanted groups, and preferentially appeared along the scaffold fibers. Compared with the RG0 and RG-1000 scaffolds, RG-400 scaffolds exhibited the highest level of newly formed bone, suggesting their superior bone regenerative performance. By 8 weeks post-implantation, the defect area in the RG-400 group was almost filled with new bone tissue. Since the critical size bone defect cannot self-heal, newly formed bone could hardly be found in the Blank group (Figure 6B). Bone volume fraction (BV/TV), trabecular number (Tb.N), trabecular thickness (Tb.Th) and trabecular separation (Tb.Sp) of the newly formed bone were quantitatively analyzed based on Micro-CT results. Both at 4 and 8 weeks, the RG-400 group exhibited a BV/TV value approximately twice that of the RG0 group, accompanied by greatly reduced Tb.Sp (Figure 6C–J). In contrast, the bone regeneration parameters of the RG-1000 group were comparable to those of RG0. These results indicate that the presence of hierarchical architectures on the scaffold fibers significantly promotes bone regeneration, but the surface features should be at a suitable size scale.

**Figure 7:**
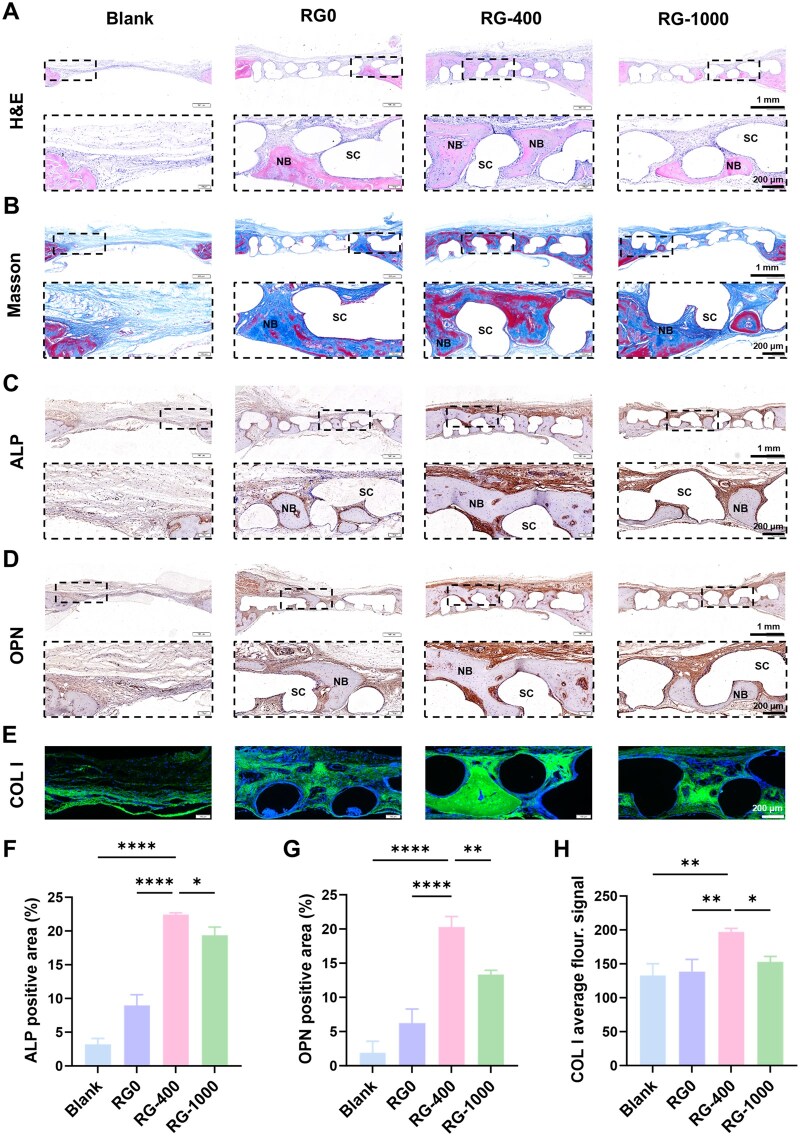
Histological evaluation of bone regeneration at 4 weeks after scaffold implantation in rat cranial defects. (**A**) Representative H&E staining images of the defect areas post-implantation. (**B**) Representative Masson staining images of the defect areas post-implantation. (**C**) Representative immunohistochemical images of ALP expression in the defects post-implantation. (**D**) Representative immunohistochemical images of OPN expression in the defects post-implantation. (**E**) Representative immunofluorescence images of COL-I expression in the defects post-implantation. (**F**) Quantitative analysis of ALP and (**G**) OPN immunohistochemical staining (*n* = 3). (**H**) Quantitative analysis of COL-I fluorescence intensity (*n* = 3). NB, new bone; SC, scaffold. (*p < 0.05, **p < 0.01, ***p < 0.001, ****p < 0.0001).

The newly formed bone within the defect area was further validated through histological evaluation using H&E and Masson staining. No new bone could be found in the Blank group, as the critical size defect exceeds the self-healing capacity of native bone (Figure 7A and B). For the 3D-printed PCL scaffold without catPG coating, only limited new bone formation was observed at the defect margins, while the central area of the defect remained filled with fibrous tissue. In contrast, the RG-400 group exhibited extensive new bone formation both along the defect boundaries and in the central region. Although some new bone formation occurred in the RG-1000 group, it was less than in the RG-400 group.

Immunohistochemical staining was further performed to verify the regulatory effect of hierarchical scaffolds on bone regeneration. As shown in Figure 7C and D, osteogenic differentiation-related markers, ALP and Osteopontin (OPN), were both highly expressed in the RG-400 group. In contrast, fewer ALP-positive and OPN-positive cells were present at the defect area in the RG0 and RG-1000 groups. These results were further confirmed by another osteogenesis-related marker, COL-I, which displayed a similar trend of elevated expression in the RG-400 group (Figure 7E and H).

## Conclusion

The repair of critical-sized bone defects remains a major clinical challenge. Although bone tissue engineering scaffolds hold great promise, their bioactivity still requires substantial improvement. Therefore, nano-/micro-hierarchical scaffolds with tunable surface architectures were developed based on a catPG. The introduction of hierarchical architectures significantly promoted scaffold bioactivity and bone regenerative performance through biophysical regulation. Scaffolds with appropriate nanoscale roughness (RG-400) provided enhanced surface contact for BMSCs, promoting filopodia extension and FA formation, thereby strengthening mechanotransduction. The increased cytoskeletal tension modulated nuclear mechanics, ultimately leading to enhanced osteogenic differentiation of BMSCs and effective repair of large defects. In contrast, low-roughness surfaces offered insufficient mechanical cues, while excessively rough interfaces (RG-1000) created physical barriers that impeded mechanotransduction signaling transmission. Overall, this study demonstrates that bone regeneration can be effectively regulated by a purely biophysical cue, namely the surface topography of the scaffold, without relying on exogenous cells, cytokines or growth factors. Meanwhile, the catPG coating in this study exhibits a substrate-independent characteristic. Its formation mainly relies on the interfacial adhesion capability of catechol groups, enabling its application to a wide range of material systems and scaffold architectures. These findings suggest a promising mechanobiological approach to repairing critical-sized bone defects by engineering scaffolds with well-defined surface architectures.

## Supplementary Material

rbag136_Supplementary_Data

## Data Availability

The data that support the findings of this study are available from the corresponding author upon reasonable request.
