# Supplementary material for: Engineered hierarchical 3D scaffold promotes bone regeneration through enhanced mechanotransduction
Source: Regen Biomater. 2026 Jun 17;13:rbag136. doi: 10.1093/rb/rbag136 (PMC13353930; doi:10.1093/rb/rbag136)
Supplement: rbag136_Supplementary_Data [file rbag136_supplementary_data.docx]

**Supporting Information**

**Engineered Hierarchical 3D Scaffold Promotes Bone Regeneration Through Enhanced Mechanotransduction**

*Dan Wu,* *Xinyu Dai, Li Xiao, Wenxuan He, Qun Li, Piaoye Ming, Yutao Zhang, Jia Li, Mingxuan Bai, Yuan Wang*, Leixiao Yu**

State Key Laboratory of Oral Diseases, National Clinical Research Center for Oral Diseases, West China Hospital of Stomatology, Sichuan University, Chengdu 610064, China

**Materials and Methods**

**Synthesis of Poly(allyl glycidyl ether) (PAGE)**

Tetra-n-octylammonium bromide (1.092 g, 1 mmol) was placed in a Schlenk flask and melted in an oil bath at 110°C under vacuum. After removing residual moisture from the flask wall, nitrogen was introduced, and 100 mL anhydrous toluene was added to dissolve the initiator. Then 10.4 mL allyl glycidyl ether (AGE, 44 mmol) was slowly added in an ice water bath, followed by the dropwise addition of triisobutylaluminum (7.2 mL, 4 mmol) to initiate polymerization. The reaction mixture was stirred overnight at room temperature. Polymerization was terminated by adding 1 mL of deionized water. Anhydrous sodium sulfate was introduced to remove residual water until the solution became clear. Toluene was removed under reduced pressure, and the residue was treated with diethyl ether to precipitate residual initiator and catalyst. The precipitate was filtered off, and ether was evaporated under reduced pressure to afford a viscous, honey-like PAGE. ^1^H-NMR (400 MHz, chloroform-d) δ = 5.88-5.75 (m, 1H, CH_2_OCH_2_C***H***=CH_2_), 5.25-5.03 (m, 2H, CH_2_OCH_2_CH=C***H***_2_), 3.90 (d, 2H, CH_2_OC***H***_2_CH=CH_2_), 3.64-3.34 (m, 7H, PG-backbone). Mn_GPC,THF_ = 8615 g/mol, Mw/Mn = 1.59.

**Synthesis of Amino-functionalized PAGE (PAmGE)**
PAGE (5 g) was dissolved into 150 mL methanol/THF (1:1). 2-Aminoethanethiol hydrochloride (19.9 g) and 2,2-dimethoxy-2-phenylacetophenone (Irgacure 651, 0.225 g) were added and stirred until dissolved. The solution was irradiated with UV light (365 nm) at room temperature overnight. The product was purified by dialysis in methanol and obtained as PAmGE after solvent removal under reduced pressure. ^1^H-NMR (400 MHz, MeOD-d4) δ = 1.85-1.97 (O-CH_2_-C***H***_2_-CH_2_-S, 2H), 2.66-2.75 (O-CH_2_-CH_2_-C***H***_2_-S, 2H), 2.81-2.91 (S-C***H***_2_-CH_2_-N, 2H), 3.10-3.19 (S-CH_2_-C***H***_2_-N, 2H), 3.83-3.51 (PG backbone), (O-C***H***_2_-CH_2_-, 2H).

**Synthesis of** **Catecholic Polyglycerol Coating Polymer (catPG)**

PAmGE (5 g) was dissolved into 100mL pH 4.8 MES aqueous buffer/methanol solution (1:1). 1-(3-Dimethylaminopropyl)-3-ethylcarbodiimide hydrochloride (EDCI, 7.525 g) and 3-(3,4-dihydroxyphenyl)-2-hydroxypropanoic acid (8.171 g) were added, and the reaction mixture was stirred at room temperature overnight. The product was purified by dialysis in methanol, and the solvent was removed under reduced pressure to yield catPG. ^1^H-NMR (400 MHz, MeOD-d4) δ = 1.63-1.76 (O-CH_2_-C***H***_2_-CH_2_-S, 2H), 2.24-2.52 (O-CH_2_-CH_2_-C***H***_2_-S, 2H), (S-C***H***_2_-CH_2_-N, 2H), (CONH-C***H***_2_-CH_2_-Ar, 2H); 2.60-2.83 (S-CH_2_-C***H***_2_-N, 2H), (CONH-CH_2_-C***H***_2_-Ar, 2H); 3.69-3.32 (PG backbone), (O-C***H***_2_-CH_2_-, 2H).

**Figures and Tables**


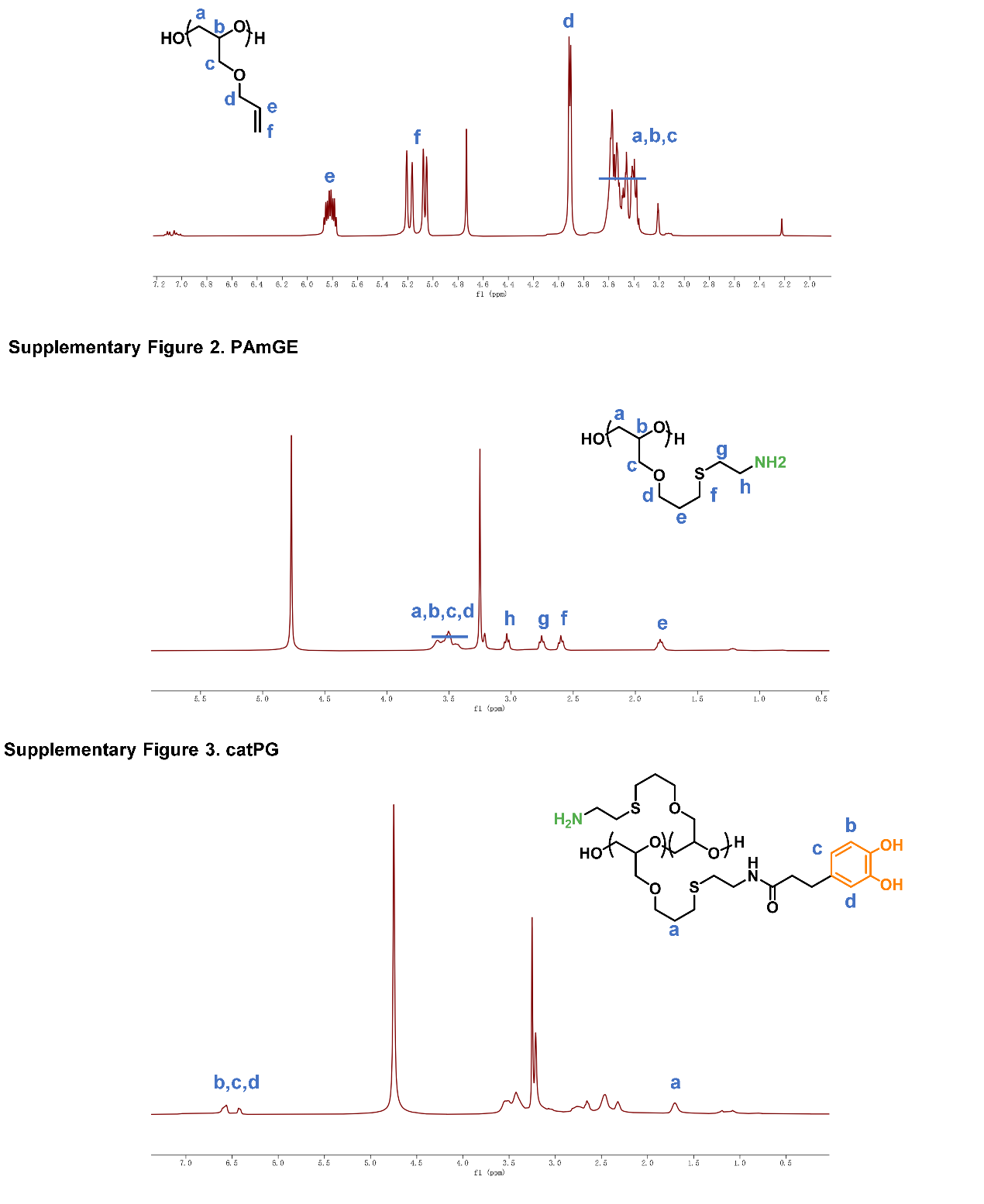


**Figure S1.** ¹H-NMR spectrum of PAGE.


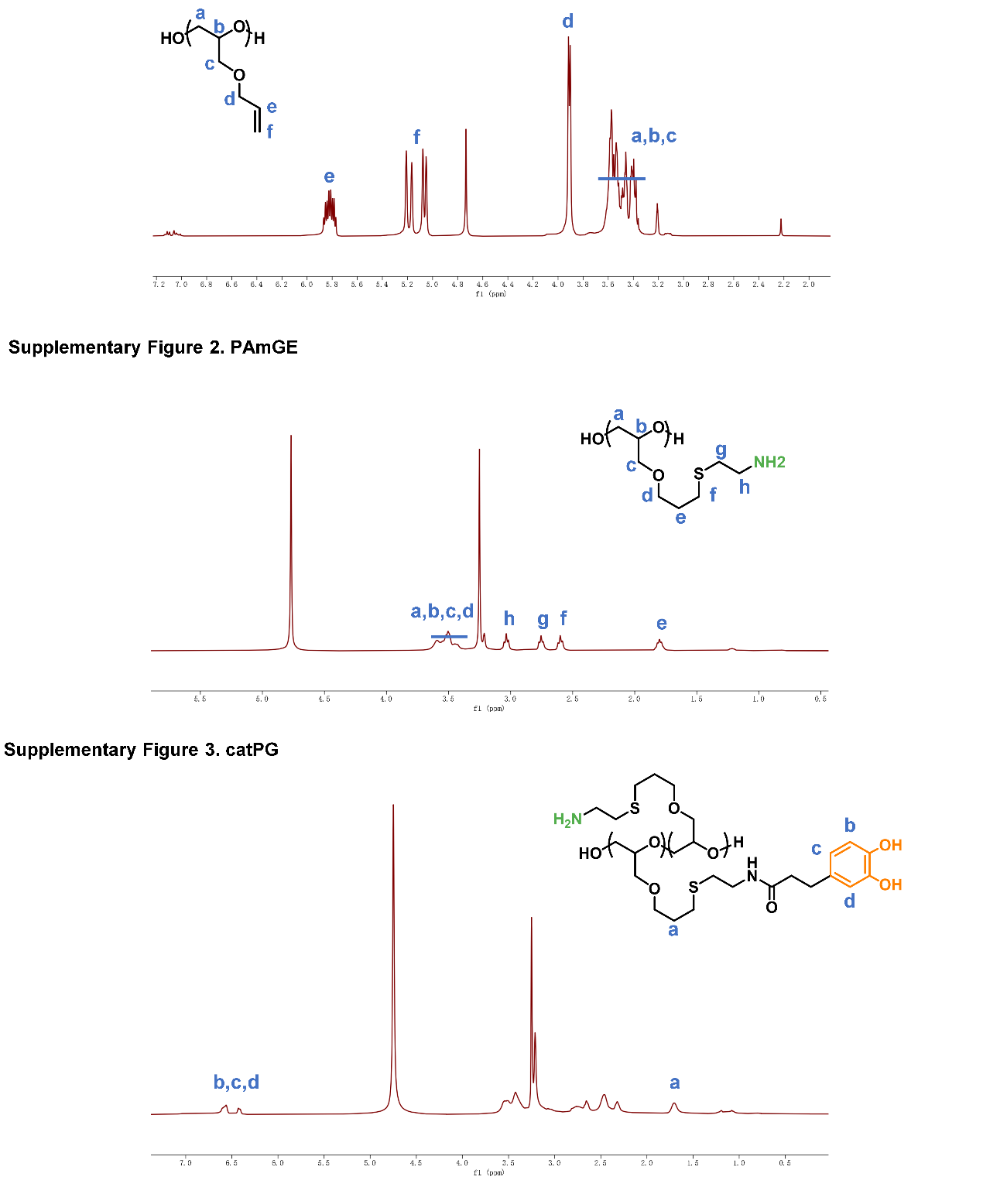


**Figure S2.** ¹H-NMR spectrum of PAmGE.


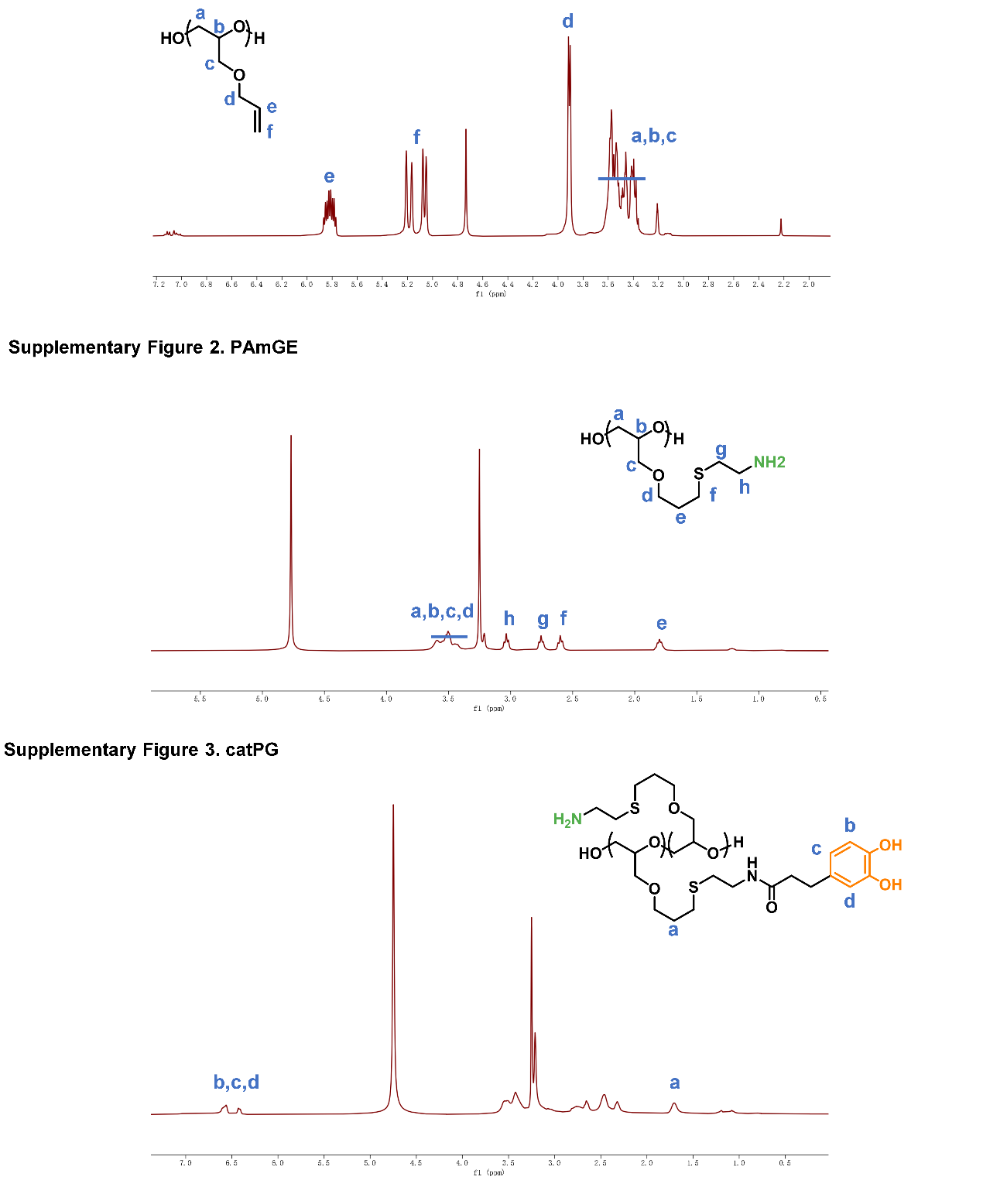


**Figure S3.** ¹H NMR spectrum of catPG.


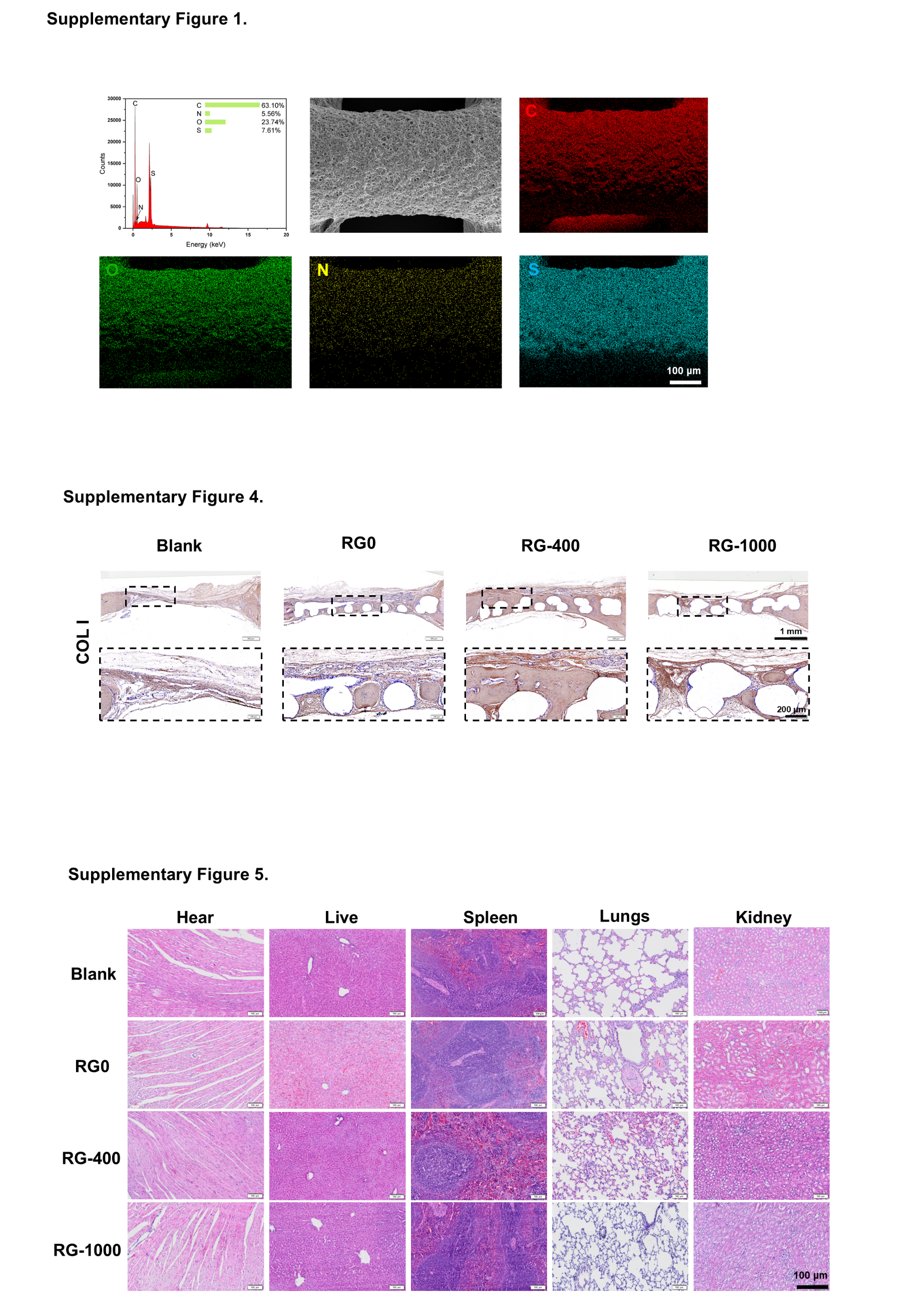


**Figure S4.** EDS elemental mapping of the surface of hierarchical scaffolds after 24 h dip-coating. The scale bar indicates 100 μm.


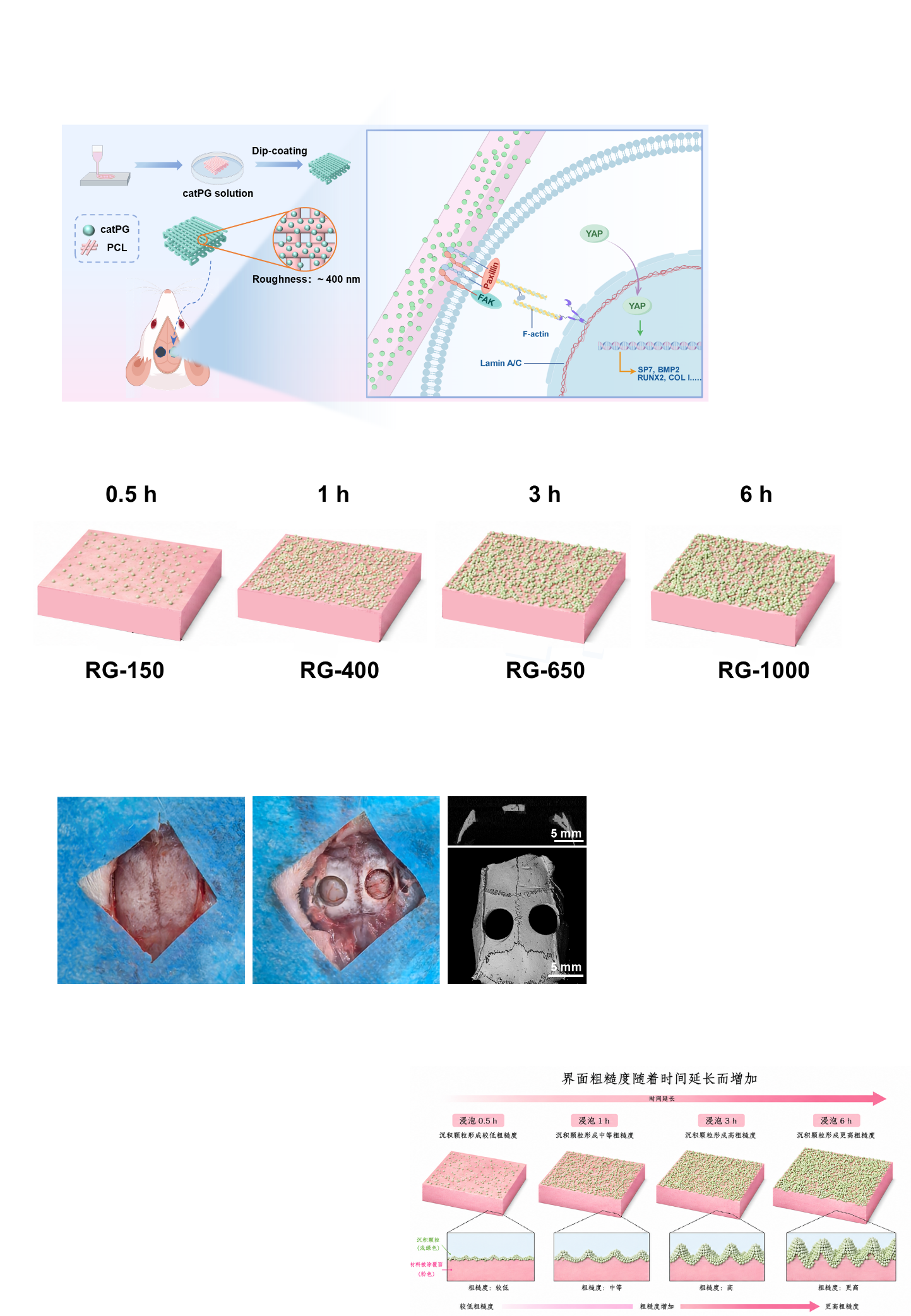


**Figure S5**. Schematic illustration of the relationship between surface roughness and interface dip-coating time.


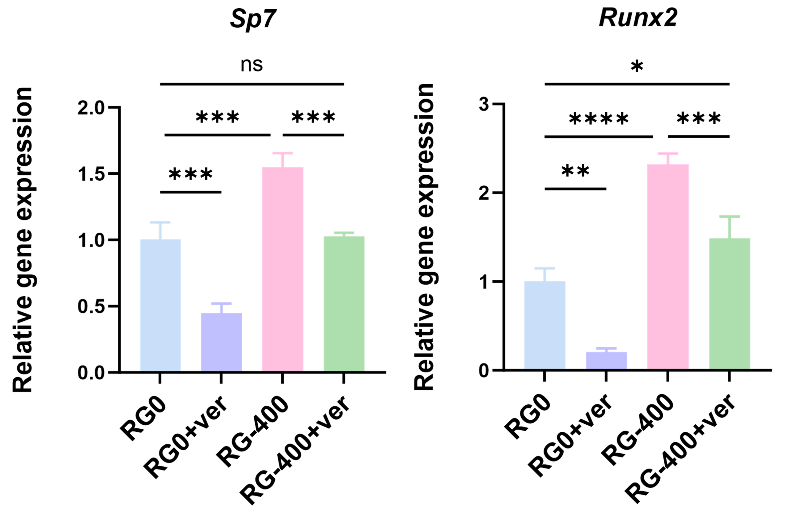


**Figure S6.** qRT-PCR analysis of *Runx2* and *Sp7* expression in BMSCs cultured on scaffolds after 3 days of Verteporfin treatment (n=3).


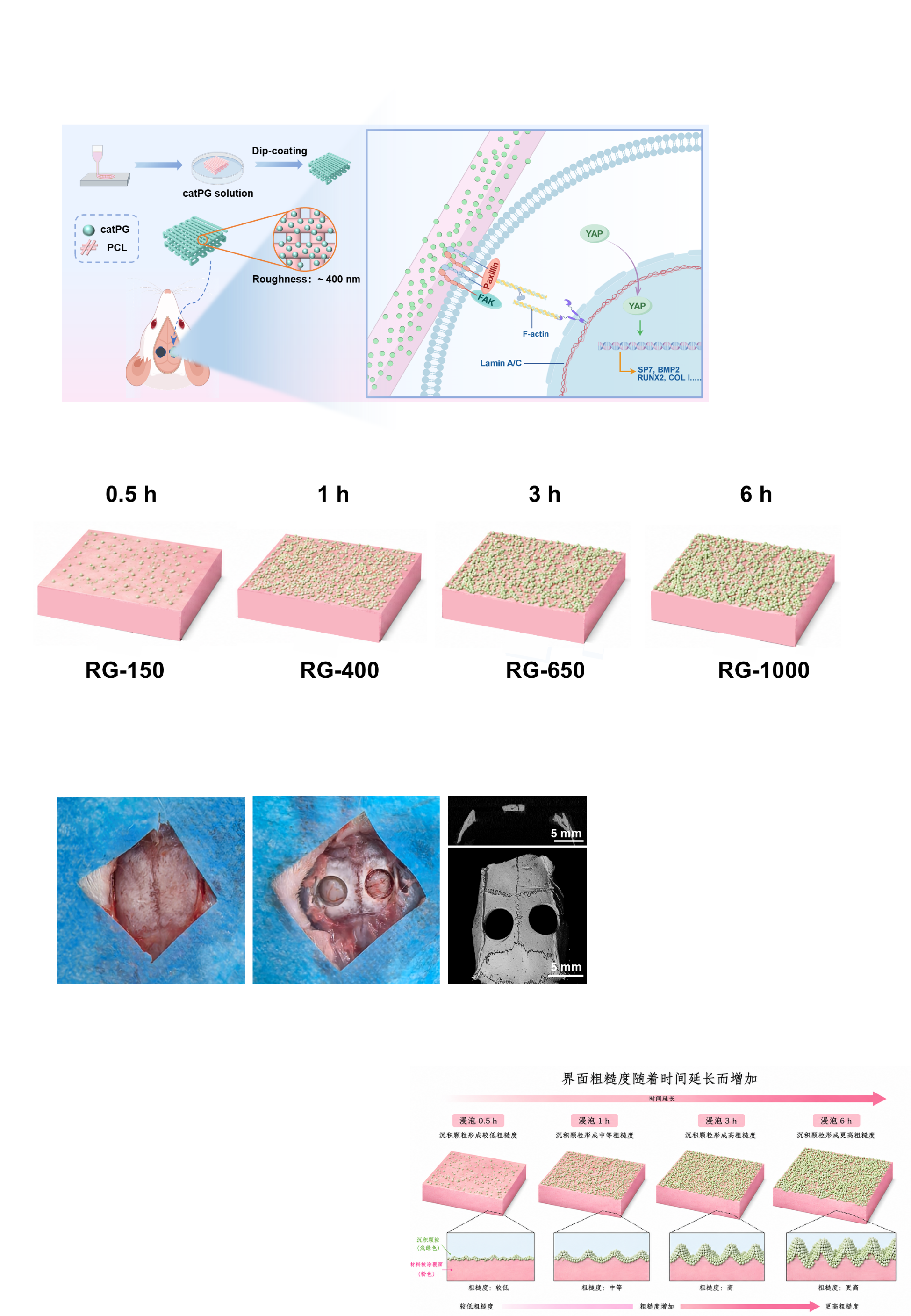


**Figure S7.** Intraoperative images of the rat calvarial defect model and representative micro-CT images acquired immediately after surgery.


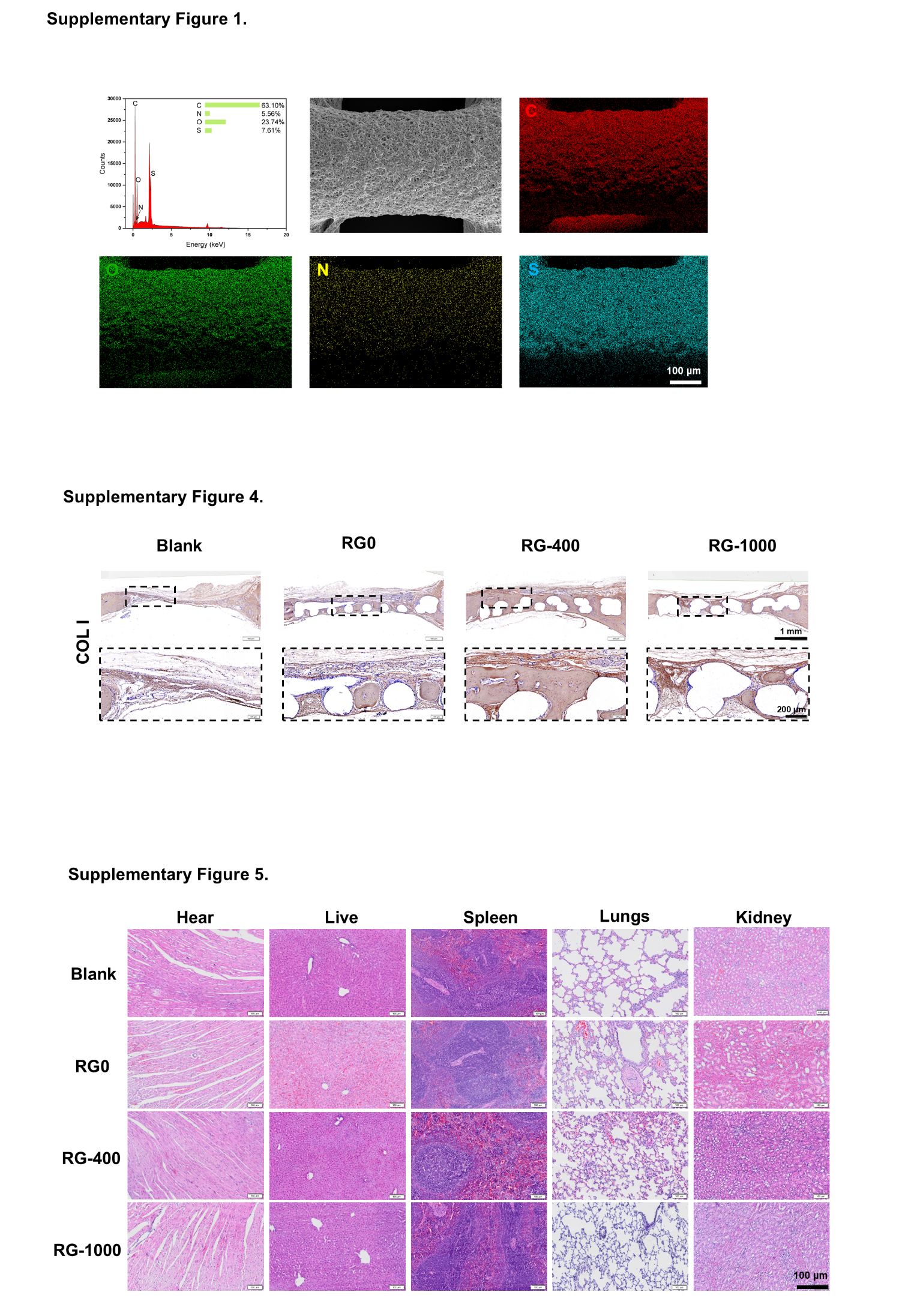


**Figure S8.** Representative immunohistochemical images of COL I expression in the defects post-implantation.


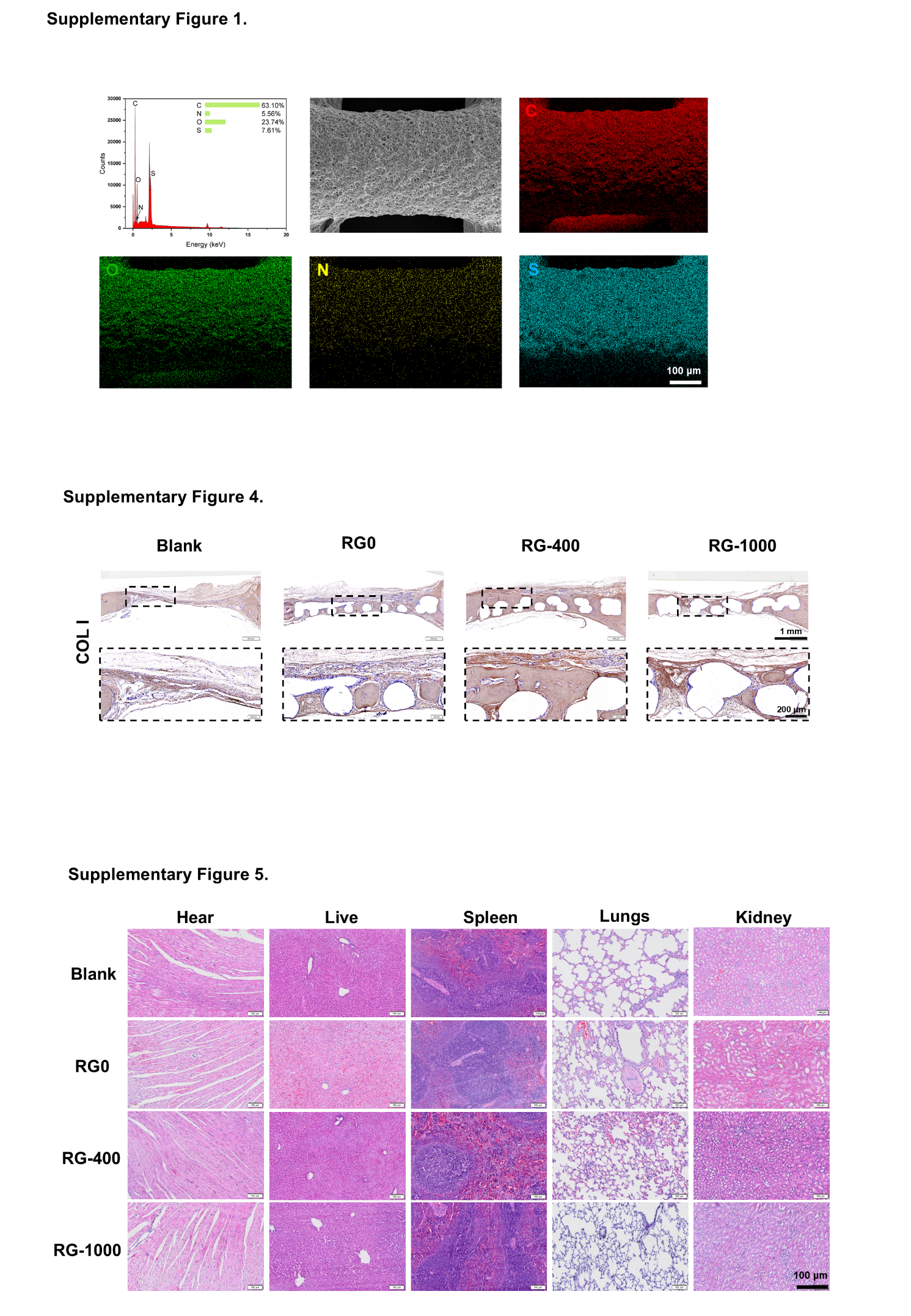


**Figure S9.** H&E staining of the heart, liver, spleen, lung, and kidney of rats.

**Table S1.** Sequences of primers used in the qPCR experiments.

| **Gene** | **Forward primer** | **Reverse primer** |
| --- | --- | --- |
| ***Bmp2*** | ACCCTTTGTATGTGGACTTCAGTGATG | CTATGGCATGGTTGGTGGAGTTCAG |
| ***Runx2*** | ATTTCAGATTCAGAGCCCAGTACCTTG | CACAGGAGGAGGACGGAGATGG |
| ***Col 1*** | TGTTGGTCCTGCTGGCAAGAATG | GTCACCTTGTTCGCCTGTCTCAC |
| ***Sp7*** | ATGGCGTCCTCTCTGCTTG | TGAAAGGTCAGCGTATGGCTT |
